# Curcumin–Lipid Interactions in PEGylated vs. Conventional Liposomes: A Combined Fluorescence and EPR Study

**DOI:** 10.3390/membranes16040137

**Published:** 2026-04-01

**Authors:** Namra Fatima, Andrzej Górecki, Anna Wiśniewska-Becker

**Affiliations:** 1 Department of Biophysics, Faculty of Biochemistry, Biophysics and Biotechnology, Jagiellonian University, 30-387 Krakow, Poland; namra.fatima@doctoral.uj.edu.pl; 2 Doctoral School of Exact and Natural Sciences, Jagiellonian University, 30-348 Krakow, Poland; 3 Department of Physical Biochemistry, Faculty of Biochemistry, Biophysics and Biotechnology, Jagiellonian University, 30-387 Krakow, Poland; andrzej.gorecki@uj.edu.pl

**Keywords:** membrane, liposome, curcumin, PEG, electron paramagnetic resonance (EPR), spin labels, fluorescence

## Abstract

Curcumin, a natural polyphenol derived from Curcuma longa, is widely recognized for its therapeutic properties. However, its clinical utility is limited because of poor solubility, rapid degradation and hence low bioavailability. To overcome these issues, nanoformulation approaches, especially PEGylated liposomes, have been explored as advanced delivery systems. PEGylation, which involves attaching polyethylene glycol (PEG) to the liposomal surface, enhances circulation time by creating a steric shield that reduces protein interactions and clearance by the mononuclear phagocyte system (MPS). However, PEG can alter lipid membrane properties, which may in turn affect curcumin’s solubility and distribution within the liposomal bilayer, ultimately reducing its loading efficiency. To ensure that PEG-modified liposomes can be effectively loaded with curcumin, we investigated curcumin–membrane interactions in saturated (DMPC) and unsaturated (POPC) liposomes, both in the presence and absence of PEG. Based on dissociation constants (K_d_) obtained from fluorescence spectroscopy measurements, we found that PEGylated DMPC liposomes exhibit the strongest binding affinity for curcumin. Fluorescence quenching experiments showed that curcumin adopts a transbilayer orientation in all membranes examined. Curcumin’s location within PEGylated and non-PEGylated liposomal membranes was further confirmed by examining its effects on membrane properties, including fluidity, polarity, and oxygen transport. These effects were investigated using electron paramagnetic resonance (EPR) spectroscopy with spin labels. The results indicate that PEG does not impose major changes on membrane properties. Curcumin, however, was found to reinforce the liposomal membranes, increase their polarity, and reduce oxygen availability. Overall, the findings suggest that liposomes, particularly those composed of PEGylated DMPC, are effective vehicles for curcumin delivery.

## 1. Introduction

Liposomes are nanoscale vesicles composed of phospholipids, known for their biocompatibility, biodegradability, and low toxicity [1]. They are extensively explored as drug delivery systems and have led to several clinical applications, particularly in cancer chemotherapy and the treatment of serious fungal infections [2]. They are also used to increase the bioavailability of antioxidant or anti-inflammatory compounds of natural origin, such as curcumin, which is otherwise poorly soluble in water [3]. Encapsulation of curcumin within liposomes has been shown to significantly improve its aqueous solubility and chemical stability, while prolonging systemic circulation and enhancing therapeutic efficacy. Additionally, liposomal delivery protects curcumin from premature degradation, promotes accumulation at target sites, and enables controlled release, making it a highly effective strategy for overcoming its pharmacokinetic limitations [3]. However, conventional liposomes are rapidly cleared from circulation through uptake by cells of the mononuclear phagocyte system (MPS), primarily in the liver and spleen. This rapid MPS uptake greatly limits their distribution to other tissues and may even exert toxic effects on MPS organs [4]. Therefore, to prolong liposome circulation time in the bloodstream, various modifications have been explored. One of the most effective strategies is PEGylation, in which polyethylene glycol (PEG) chains are covalently attached to phosphatidylethanolamine (PE) lipids incorporated into the liposomal membrane [5]. Another critical challenge in liposome technology is achieving high drug loading efficiency. The extent to which liposomes encapsulate therapeutic compounds is largely governed by the biophysical and structural properties of their lipid bilayers, including fluidity, micropolarity, and internal free volume. These membrane characteristics can be modified through the incorporation of specific lipid additives [6]. In this regard, PEGylated lipids have been shown to influence bilayer organization, reduce interfacial hydration, and enhance overall vesicle stability [7,8,9]. Also, curcumin’s effects on various membrane properties were investigated in liposomes [10]. In our previous study [11], we examined in detail the effects of curcumin on membrane properties, such as fluidity, polarity, and phase transition. The main aim of this research was to determine curcumin’s solubility and orientation within the liposomal membrane. The results indicated a transbilayer orientation perpendicular to the membrane surface, with two curcumin molecules likely forming dimers through hydrogen bonding within the bilayer. These findings were compared with the orientation and effects of carotenoids on membranes and discussed in terms of possible antioxidant properties. Interestingly, curcumin (10 mol %) exerted a rigidifying effect on 1,2-dimyristoyl-*sn*-glycero-3-phosphocholine (DMPC) and 1,2-distearoyl-*sn*-glycero-3-phosphocholine (DSPC) membranes, while strongly increasing polarity both in the polar headgroup region and at the membrane center. The first effect was like that observed for carotenoids, but the latter was not, raising questions about curcumin’s protective role against lipid peroxidation.

Considering that curcumin is located within the lipid membrane and that PEGylation of liposomes extends circulation time, reduces immune clearance, and may alter lipid membrane properties, we focused in the present work on curcumin-containing PEGylated liposomes as potential carriers for in vivo curcumin delivery. In particular, this study aims to elucidate how liposome PEGylation affects lipid–curcumin interactions and curcumin loading efficiency. To achieve this, we employed two complementary approaches. First, leveraging the intrinsic fluorescence of curcumin (emission at 460–550 nm depending on the local environment) [12], we applied fluorescence spectroscopy to directly estimate the binding affinity of curcumin to different liposomes (saturated DMPC vs. unsaturated 1-palmitoyloyl-2-oleoyl-*sn*-glycero-3-phosphocholine POPC, PEGylated vs. non-PEGylated). Second, we investigated the location of curcumin within the bilayer indirectly by analyzing its fluorescence quenching in the presence of paramagnetic agents positioned at defined depths along lipid acyl chains. Finally, using electron paramagnetic resonance (EPR) spectroscopy and lipid spin labels, we evaluated the impact of curcumin on membrane fluidity, polarity, and oxygen transport in PEGylated and non-PEGylated DMPC and POPC liposomes. By integrating these approaches, our work aims to elucidate the mechanistic impact of PEGylation on drug–liposome interactions and contribute to the rational design of more effective liposomal carriers for poorly soluble drugs such as curcumin.

## 2. Materials and Methods

### 2.1. Liposome Preparation

DMPC, POPC, 14:0-PEG2000-PE (1,2-dimyristoyl-sn-glycero-3-phosphoethanolamine-N-[methoxy(polyethylene glycol)-2000]), 18:0-PEG2000-PE (1,2-distearoyl-sn-glycero-3-phosphoethanolamine-N-[methoxy(polyethylene glycol)-2000]), curcumin (1,7-bis(4-hydroxy-3-methoxyphenyl)1,6-heptadiene-3,5-dione), spin labels (5- and 16-doxyl stearic acid (5- and 16-SASL) and 1-palmitoyl-2-stearoyl-(n-doxyl)-*sn*-glycero-3-phosphocholine n-PC, where n = 5 or 16) and T-PC (1,2-dipalmitoyl-*sn*-glycero-3-phospho(tempo)choline) were purchased from Merck, Warsaw, Poland. Multilamellar liposomes for EPR measurements were prepared of DMPC or POPC. If required, curcumin was added in the final concentration of 10 mol %, and PEGylated PE in the final concentration of 7 mol %. To match the lengths of host lipids with PEGylated ones, 14:0-PEG2000-PE was added to DMPC, and 18:0-PEG2000-PE to POPC liposomes. Two types of liposomes were employed in this study: multilamellar vesicles for EPR measurements and unilamellar vesicles for fluorescence experiments. Unilamellar liposomes are the most commonly used carriers for drug and bioactive compound delivery, as they can be readily downsized to a desired diameter and exhibit greater size homogeneity than multilamellar systems [13]. However, multilamellar liposomes provide a high concentration of lipid membranes, which is required to obtain EPR signals of sufficient intensity from incorporated spin labels. Simply increasing the spin label concentration beyond the recommended 1 mol % is not advisable, as spin labels may perturb membrane properties and cause spectral broadening due to Heisenberg exchange interactions between closely spaced spins [14]. In fluorescence measurements, such constraints do not apply. Moreover, when curcumin is introduced from an ethanol solution into preformed liposomes, equilibration occurs substantially faster in unilamellar vesicles than in multilamellar ones, further justifying their use in fluorescence experiments.

The liposomes were prepared by the thin film hydration method [15]. Briefly, for EPR measurements, chloroform solutions of lipids (DMPC or POPC with or without PEGylated phosphatidylethanolamine) together with spin labels (1 mol %) were combined. If applicable, ethanol solution of curcumin was added to the chloroform mixture of lipids. The final total amount of lipids was 2.5 µmol. The solvent was then removed under a stream of nitrogen, and the resulting lipid film formed at the bottom of the test tube was further dried under reduced pressure (~0.1 mm Hg) for 12 h. The dried film was hydrated with 0.5 mL phosphate-buffered saline (pH 7.4) at a temperature well above the lipid phase transition temperature (T_m_) and vigorously vortexed. The resulting multilamellar liposome suspension was subjected to five freeze–thaw cycles, followed by centrifugation at 14,000× *g* for 15 min at 4 °C. The final pellet was collected and used for EPR measurements. Unilamellar liposomes for fluorescence measurements were prepared by extruding multilamellar liposome suspensions—composed of DMPC or POPC and, when applicable, the appropriate PEGylated phosphatidylethanolamine—through polycarbonate membranes with a pore size of 100 nm using a mini-extruder (Avanti Polar Lipids, Alabaster, AL, USA). Curcumin was subsequently incorporated by adding it from a concentrated ethanol solution to the preformed unilamellar liposomes.

### 2.2. Fluorescence Measurements

#### 2.2.1. Curcumin–Liposome Dissociation Constant

Dissociation constant values (K_d_) were calculated based on titration curves obtained by adding a concentrated ethanol solution of curcumin to unilamellar liposome suspensions of increasing lipid concentration (in the range of 0.0019 to 2 mM). The final concentration of curcumin was 2 µM. Fluorescence intensity of curcumin was measured using a spectrofluorimeter (PerkinElmer LS 55 Luminescence Spectrometer, PerkinElmer Inc., Shelton, CT, USA) with a quartz cuvette of 1 mL volume. The excitation wavelength was 425 nm, and the maximum emission was observed at 525 nm. The data from three independent experimental series were globally fitted to a single-site binding model using the DynaFit program (version 4.09.019; BioKin, Watertown, MA, USA) [16]. The global analysis was performed separately for POPC and DMPC liposomes, each under two conditions: with and without PEG.

#### 2.2.2. Curcumin Fluorescence Quenching by n-SASL

Unilamellar liposomes of POPC, POPC-PEG, DMPC and DMPC-PEG containing 15 mol % of 5- or 16-SASL were prepared, and curcumin was then added from a concentrated ethanol solution to achieve a final concentration of 2 µM. The samples were then allowed to equilibrate on a shaker (PRO-BLOT Rocker 25, Labnet International Inc., Edison, NJ, USA) for 0.5 and 1 h. Liposomes without spin labels served as a control. Curcumin fluorescence intensity was measured as described above—using a PerkinElmer LS 55 Luminescence Spectrometer with a quartz cuvette of 1 mL volume. The excitation wavelength was 425 nm, and the maximum emission was observed at 525 nm.

### 2.3. EPR Measurements

#### 2.3.1. Continuous Wave (CW)

Continuous-wave (CW) EPR measurements were carried out using a Bruker EMX spectrometer (Bruker BioSpin GmbH, Rheinstetten, Germany) quipped with a temperature control unit (EMX ER 4141 VT, Bruker BioSpin GmbH, Rheinstetten, Germany). Suspensions of multilamellar liposomes containing 1 mol % spin label were loaded into gas-permeable TPX capillaries (inner diameter 0.7 mm) and placed in an EPR dewar insert within the resonant cavity of the spectrometer. Prior to measurements, samples were thoroughly deoxygenated by purging with nitrogen gas for approximately 10 min; nitrogen flow was also used to maintain temperature control. For the determination of lipid order parameters and rotational correlation times, EPR spectra were recorded at 20, 25, and 37 °C for POPC, and at 25 and 37 °C for DMPC. For polarity measurements, expressed as the maximum hyperfine splitting (2A_z_), samples were frozen to 120 K (−153 °C) using liquid nitrogen vapor [11]. Exemplary EPR spectra of all used spin labels together with applied formulas are presented in the Supplementary Materials.

#### 2.3.2. Saturation Recovery (SR)

Saturation recovery (SR) EPR measurements were performed using a home-built pulse spectrometer [17] operating at X-band and equipped with a 1 mm loop-gap resonator [18]. Liposome samples were prepared and loaded into TPX capillaries and positioned within the resonant cavity of the spectrometer, following the same procedure as used for CW EPR measurements. For determination of the oxygen transport parameter (OTP), the oxygen concentration in the samples was controlled by equilibration with the same gas used for temperature regulation, either air or nitrogen [19]. Spin–lattice relaxation times (T_1_) of the spin labels were obtained by analyzing the saturation recovery signals of the central EPR line. SR curves were digitized using 2048 points at acquisition rates adjusted according to the gas atmosphere and relaxation rate. The resulting SR curves were fitted with single-exponential functions using the Eleana software package (https://sourceforge.net/projects/eleana/ (ver. 4.2, accessed on 28 April 2025, 16 May 2025, 16 July 2025 and 17 July 2025).

## 3. Results

### 3.1. Effect of PEGylation on Curcumin Binding to DMPC and POPC Liposomes

**Figure 1:**
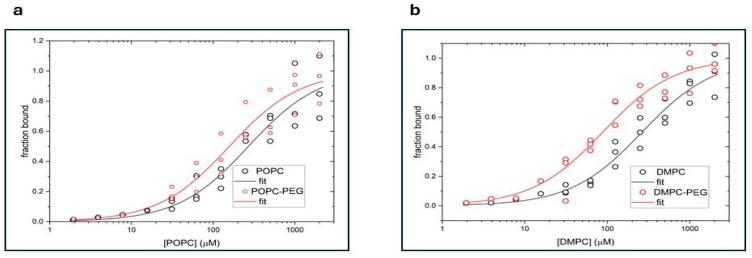
Fluorescence measurements of curcumin binding to liposomes composed of (**a**) POPC (black circles) or POPC-PEG (red circles) and (**b**) DMPC (black circles) or DMPC-PEG (red circles). The phosphatidylcholine concentration is shown on the *x*-axis, and the fraction of bound curcumin on the *y*-axis. The solid lines represent the best fits obtained using the DynaFit software.

Figure 1 presents titration curves obtained for POPC and POPC-PEG (Figure 1a), and DMPC and DMPC-PEG (Figure 1b) liposomes. Curcumin fluorescence intensity was measured for increasing concentrations of lipids. The data were globally fitted to a single-site binding model using the DynaFit program and dissociation constants (K_d_) for curcumin with various liposomes were calculated (Table 1).

K_d_ values of curcumin are comparable for POPC and DMPC membranes. However, for both types of liposomes, PEGylation markedly enhances curcumin binding, increasing the binding affinity by approximately threefold in DMPC liposomes and by about 1.7-fold in POPC liposomes. These results indicate that PEGylation enhances curcumin–lipid interactions more strongly in DMPC membranes than in POPC membranes.

### 3.2. Quenching of Curcumin Fluorescence by Lipid Spin Labels

**Figure 2:**
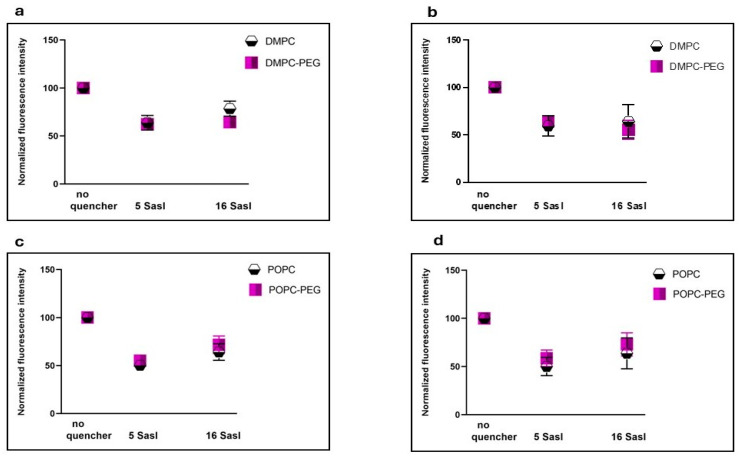
Quenching effect of stearic acid spin labels (SASL) having quenching moieties attached at two different positions (5 and 16) on the fluorescence of curcumin incubated with DMPC, DMPC-PEG (**a**,**b**), POPC, and POPC-PEG (**c**,**d**) liposomes for 0.5 h (**left panel**) and 1 h (**right panel**).

To assess the location of curcumin within the liposomal membranes, its fluorescence intensity was measured in the presence of 5- and 16-SASL and compared with a control sample without the quenchers (Figure 2). In 5-SASL, the paramagnetic nitroxide moiety responsible for fluorescence quenching is in the region close to the membrane surface, whereas the free radical moiety of 16-SASL is embedded deep within the membrane center [20,21]. In all the membranes studied, curcumin quenching was significant at both probe positions, although some differences were observed. In non-PEGylated DMPC, quenching by 16-SASL was slightly weaker than by 5-SASL (78% vs. 63%), whereas PEGylated DMPC exhibited comparable quenching by 5- and 16-SASL (62% and 64%). In POPC membranes, both PEGylated and non-PEGylated, quenching was modestly stronger near the fifth carbon than at the bilayer center (55% vs. 71% and 46% vs. 58%, respectively).

### 3.3. Effect of Curcumin on Lipid Mobility in PEGylated and Non-PEGylated Liposomes

#### 3.3.1. Polar Headgroup Region

**Figure 3:**
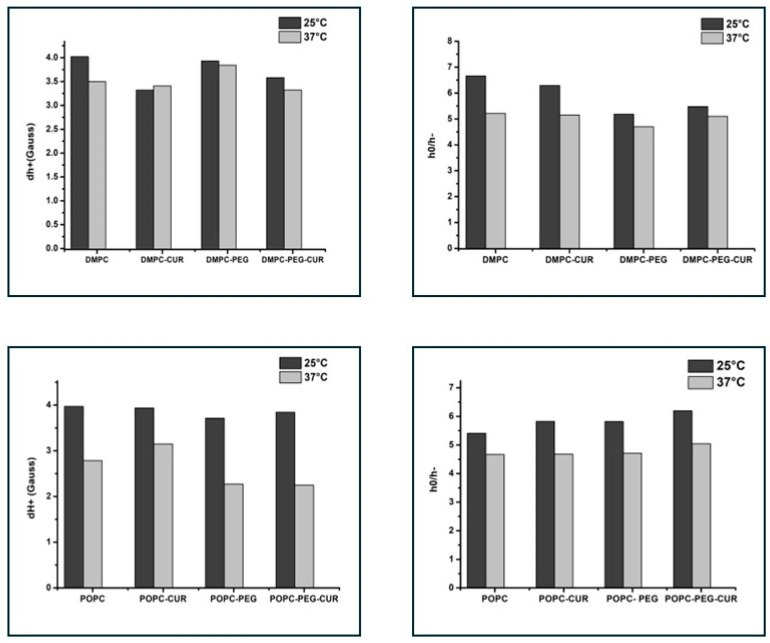
EPR spectral parameters of T-PC spin probe measured for DMPC, DMPC-PEG, POPC and POPC-PEG membranes in the presence and absence of 10 mol % curcumin at 25 °C and 37 °C. *h*_0_/*h*_−_ is a ratio of the height of central and high-field peaks and dh+ is the peak-to-peak width of the low-field line. Because of the sharpness of the low-field line for T-PC (see Figure S1), the accuracy of measurements of dh+ is ±0.1 G.

To investigate the membrane properties in the polar headgroup region, the T-PC spin label was used. The parameters obtained from the EPR spectra of T-PC at 25 °C and 37 °C, the peak-to-peak width of the low-field line (dh+) and the ratio of the height of central and high-field peaks (*h*_0_/*h*_−_) (Supplementary Materials, Figure S1), give information about the headgroup motional freedom [11]. The increase in motional freedom leads to a decrease in these parameters, as evidenced by the comparison of values at 25 °C and 37 °C (Figure 3). In DMPC membranes, both spectral parameters have smaller values in the presence of curcumin, especially at 25 °C (dh+ is reduced by about 20% and *h*_0_/*h*_−_ by about 5%), indicating an increase in lipid headgroups’ mobility, and the difference is smaller in the PEGylated membranes (6% and none, respectively). In POPC membranes, both PEGylated and non-PEGylated, the effect is negligible. The results suggest that in the presence of curcumin, the polar headgroups of DMPC molecules are more separated from each other and have more motional freedom than in pure DMPC membrane, while PEG reduces this effect.

#### 3.3.2. Alkyl Chain Region

Membrane fluidity may be described in terms of static or dynamic parameters. The static one, order parameter S, reflects the segmental order parameter of the hydrocarbon chain segment to which the nitroxide fragment is attached [22]. It was calculated based on spectral parameters of 5- and 16-PC spin labels according to Marsh [23] (Supplementary Materials, Figure S1). As shown in Table 2, curcumin increases acyl chain order at both probe positions in DMPC membranes, particularly at 25 °C, with S values rising from 0.63 to 0.66 for 5-PC and from 0.13 to 0.18 for 16-PC. A similar ordering effect is observed in PEGylated DMPC, where S increases from 0.64 to 0.69 (5-PC) and from 0.13 to 0.17 (16-PC). Notably, PEG alone also induces ordering, but only near the membrane headgroup region, as indicated by a 0.01 increase in the 5-PC S parameter compared with non-PEGylated DMPC. In POPC membranes, the ordering effect of curcumin is weaker and predominantly confined to the headgroup region, with S increasing from 0.635 to 0.66 for 5-PC and from 0.125 to 0.135 for 16-PC. In PEGylated POPC, curcumin affects only the headgroup region (S parameter of 5-PC increases from 0.645 to 0.675), while PEG alone produces a similar modest ordering effect as in DMPC. At 37 °C, curcumin-induced ordering is detectable only at the 5-PC position in all membranes studied.

**Figure 4:**
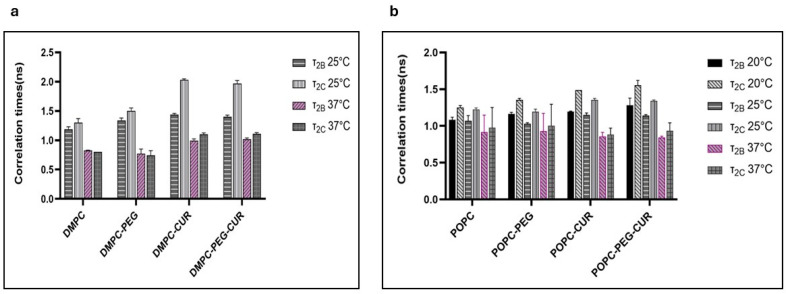
Rotational correlation times τ_2B_ and τ_2C_ of 16-PC in (**a**) DMPC (at 25 and 37 °C) and (**b**) POPC (at 20, 25 and 37 °C) membranes in the presence and absence of 10 mol % curcumin and/or 7 mol % PEG.

The relatively fast motion of the 16-PC spin label produces more isotropic spectra, enabling the calculation of dynamic parameters, specifically the rotational correlation times τ_2B_ and τ_2C_, using the formulas provided by Berliner [24]. As shown in Figure 4, curcumin clearly increases the rotational correlation times of 16-PC in DMPC and POPC membranes, both PEGylated and non-PEGylated. For example, in DMPC at 25 °C, curcumin increases τ_2B_ of 16-PC from 1.19 to 1.44 ns, and τ_2C_ from 1.3 to 2.03 ns. In POPC, the effect is less pronounced but still significant—at 25 °C curcumin increases τ_2B_ of 16-PC from 1.07 to 1.15 ns, and τ_2C_ from 1.22 to 1.33 ns. Additionally, membranes containing curcumin show a greater difference between τ_2B_ and τ_2C_, (0.59 vs. 0.11 ns for DMPC and 0.19 vs. 0.15 ns for POPC at 25 °C) indicating that in the membrane center, the motion of lipid acyl chains is not only slower but also more anisotropic compared to membranes without curcumin. A discernible ordering effect of PEG alone is observed only in DMPC, with τ_2_B increasing from 1.19 to 1.34 ns and τ_2_C from 1.30 to 1.50 ns at 25 °C upon PEG addition.

### 3.4. Effect of Curcumin on Polarity Profiles of PEGylated and Non-PEGylated Liposomes

**Figure 5:**
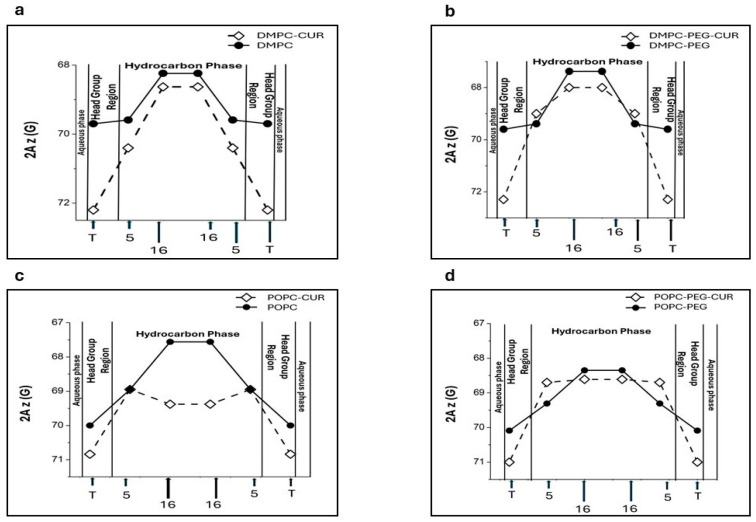
Polarity profiles across DMPC (**a**), DMPC-PEG (**b**), POPC (**c**) and POPC-PEG (**d**) membranes in the presence and absence of 10 mol % curcumin. Higher 2A_z_ values (shown inverted in the plot) indicate increased polarity. Approximate locations of the nitroxide moieties of spin labels are indicated by arrows.

The 2A_z_ parameter (z-component of the hyperfine interaction tensor) obtained from the EPR spectra of spin labels in frozen suspensions of liposomes is sensitive to the local polarity [25]. Higher values of this parameter indicate higher polarity. Based on this dependence, polarity profiles across lipid bilayer membranes can be obtained [11,15,25]. Figure 5 depicts polarity profiles obtained for DMPC (Figure 5a), DMPC-PEG (Figure 5b), POPC (Figure 5c) and POPC-PEG (Figure 5d) in the presence and absence of 10 mol % curcumin. Curcumin induces substantial changes in all investigated membrane systems. In DMPC, curcumin markedly enhances water penetration into the polar headgroup region, as reported by T-PC, where 2A_z_ increases by approximately 2.5 G in the presence of curcumin. A more modest increase in water penetration is also observed in the alkyl chain region at the 5th and 16th carbon positions, with corresponding 2A_z_ increases of 0.65 G and 0.39 G, respectively (Figure 5a). PEG alone does not significantly modify the polarity profile of DMPC, although a slight decrease in polarity is observed at the center of the PEGylated membrane (Figure 5b). In PEGylated DMPC membranes, curcumin produces a larger increase in polarity than in non-PEGylated membranes, but this effect is confined to the membrane center, where the difference in 2A_z_ values reaches 0.62 G compared with 0.39 G in the absence of PEG. Notably, in PEGylated DMPC membranes, curcumin does not increase polarity near the fifth carbon position. In the POPC membrane, the effect of curcumin is also significant, but different than in DMPC (Figure 5c,d). First, the curcumin-induced increase in polarity within the headgroup region is weaker in both PEGylated and non-PEGylated POPC membranes compared to DMPC (2A_z_ of T-PC increases by approximately 0.9 G in the presence of curcumin). Second, a strong increase in polarity is observed in the membrane center (2A_z_ of 16-PC increases by approximately 1.9 G in the presence of curcumin, Figure 5c). This effect is especially noticeable because the POPC membrane is inherently more hydrophobic at the center than DMPC. PEG alone does not significantly affect the polarity of the headgroup region; however, unlike in DMPC, it markedly reduces the hydrophobicity barrier at the center of POPC membranes, where 2A_z_ increases by 0.85 G upon PEGylation. In this region of the membrane, PEG also diminishes the effect of curcumin. In POPC membranes, the difference in 2A_z_ values with and without curcumin is approximately 1.9 G, whereas in PEGylated POPC it is only 0.3 G.

### 3.5. Effect of Curcumin on Oxygen Transport Parameters of PEGylated and Non-PEGylated Liposomes

The saturation recovery (SR) EPR technique is used to determine the spin–lattice (T_1_) relaxation times of paramagnetic probes, such as nitroxide spin labels incorporated into lipid membranes [26]. The T_1_ relaxation time reflects how quickly the spin system returns to equilibrium after excitation. Molecular oxygen strongly shortens the T_1_ of spin labels because of efficient Heisenberg exchange during collisions between oxygen molecules and the spin label. This oxygen-induced effect on T_1_ is much larger than changes caused by molecular motion of the probe itself. As a result, SR EPR provides a sensitive means of probing oxygen dynamics within membranes. By comparing T_1_ values measured in samples equilibrated with atmospheric air and with nitrogen, the oxygen-dependent contribution to spin–lattice relaxation can be isolated. This contribution reflects the frequency of collisions between the spin label and oxygen molecules and is therefore related to the oxygen diffusion–concentration product, D_0_[O_2_], within the membrane [27]. To quantify this effect, Kusumi et al. [28] introduced the oxygen transport parameter (OTP), W(x), defined as:

W(x) = T_1_^−1^ (air, x) − T_1_^−1^ (N_2_, x),

where T_1_ (air, x) and T_1_ (N_2_, x) are the spin–lattice relaxation times of the nitroxide at a specific depth x in the membrane under air and nitrogen atmospheres, respectively. The OTP thus provides a direct measure of the collision rate between the spin probe and molecular oxygen at depth x. This collision rate is proportional to both the local oxygen concentration, C(x), and the local oxygen diffusion coefficient, D(x), in the membrane.

**Figure 6:**
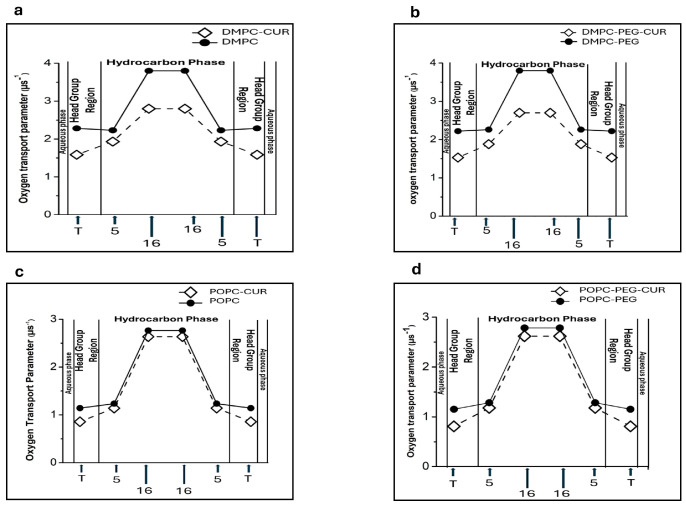
Profiles of oxygen transport parameters across DMPC (**a**), DMPC-PEG (**b**), POPC (**c**), and POPC-PEG (**d**).

Figure 6 shows the profiles of OTP obtained for DMPC (Figure 6a), DMPC-PEG (Figure 6b), POPC (Figure 6c) and POPC-PEG (Figure 6d) in the presence and absence of 10 mol % curcumin. Membranes lacking curcumin exhibit distinct changes in the oxygen transport parameter (OTP) along the lipid alkyl chains. All investigated membranes, both PEGylated and non-PEGylated, exhibit significant oxygen permeability in their central regions (16-PC), whereas oxygen penetration is reduced in the polar headgroup region (T-PC) and near this region (5-PC). The difference in OTP between T-PC and 16-PC is about 1.7 µs^−1^ in all membranes studied. The presence of curcumin significantly alters these profiles, leading to a pronounced decrease in the oxygen transport parameter at the membrane center. The effect is stronger in DMPC, both PEGylated and non-PEGylated, where OTP is reduced by about 1.4 µs^−1^ compared to the membrane without curcumin. In the polar headgroup region, where OTP is already low in pure membranes, the presence of curcumin leads to a further reduction of approximately 0.8 μs^−1^. This effect is again more pronounced in DMPC than in POPC. PEG alone does not significantly influence oxygen penetration into the membranes, as the OTP profiles of PEGylated and non-PEGylated DMPC and POPC membranes are nearly identical.

## 4. Discussion

Among different pharmaceutical nanocarriers, PEGylated liposomes are quite commonly used. Incorporating a polymer to form a corona around liposomes significantly prolongs their circulation time in the bloodstream by reducing their clearance by the mononuclear phagocyte system (MPS) [9]. However, the exact mechanism of this prevention is not completely understood. Moreover, growing evidence indicates that the polymeric corona is not entirely inert, and PEGylated nanoparticles can engage with the biological environment through Van der Waals forces, hydrophobic interactions, and hydrogen-bond formation [29]. PEG presence may also impact liposome interactions with the cell membrane after delivery and influence their subsequent biological fate [30]. For example, the fusion of liposomes with cell membranes may be influenced by PEG [31]. Studies on PEG effects on liposome membranes may help to understand more about these interactions. In this case, liposomes can be studied not only as nanocarriers but also can serve as models of cell membranes. Another feature of PEG is its ability to enhance the partitioning of hydrophobic compounds into lipid membranes. A PEG corona around liposomes has been reported to enhance the binding affinity of these compounds for lipid bilayers [7]. Therefore, PEGylation may increase the loading efficiency of liposomes for hydrophobic molecules. In the present work, we focused on curcumin-loaded liposomes and examined the potential effects of PEG on curcumin–liposome interactions and loading efficiency.

### 4.1. Fluorescence Experiments

**Figure 7:**
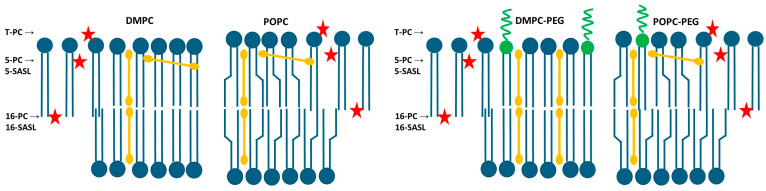
Schematic representation of the approximate location of curcumin (orange bars) in non-PEGylated (**left panels**) and PEGylated (**right panels**) DMPC and POPC membranes. Blue shapes represent lipid molecules while green shapes represent PEGylated lipids. The positions of nitroxide moieties of lipid spin labels used in the study (T-PC, 5-PC, 5-SASL, 16-PC and 16-SASL) are indicated by red stars and showed by arrows. The *cis* double bond between the 9th and 10th carbon atoms in the oleyl chain of POPC is depicted as a chain bend. Curcumin preferentially adopts a transbilayer orientation, with a fraction of molecules residing closer to the membrane surface. In PEGylated DMPC membranes, curcumin is more uniformly distributed across the bilayer.

First, taking advantage of curcumin’s intrinsic fluorescence, we examined its interaction with both PEGylated and non-PEGylated DMPC and POPC liposomes. The calculated dissociation constant (K_d_) values were markedly lower for PEGylated liposomes, particularly for PEGylated DMPC, compared with their non-PEGylated counterparts. The K_d_ values decreased from 263 µM for DMPC to 88 µM for PEGylated DMPC and from 270 µM for POPC to 156 µM for PEGylated POPC. This indicates that PEG enhances curcumin binding, and potentially its partitioning, into lipid membranes, with the length of the PEG lipid anchor also appearing to play a role. A similar effect of increased partitioning in PEGylated membranes was observed previously for hydrophobic porphyrin p-THPP [7,9]. However, determining curcumin’s binding affinity to membranes does not provide information about its spatial location within the lipid bilayer. Using EPR spectroscopy, we previously demonstrated that in non-PEGylated liposomes composed of saturated lipids curcumin adopts a transbilayer orientation [11]. In the present study, fluorescence quenching was employed to compare the location of curcumin in PEGylated and non-PEGylated membranes. The vertical position of curcumin within lipid membranes was assessed using nitroxide-labeled stearic acids (5-SASL and 16-SASL), which are well-established and efficient fluorescence quenchers [9,21,32]. Given the chemical structure and length of the curcumin molecule, as well as the thickness of the lipid bilayer, efficient quenching at both the 5th and 16th carbon positions can occur only if curcumin is oriented approximately perpendicular to the membrane surface and aligned with the lipid acyl chains (Figure 7).

In PEGylated DMPC, after 30 min of incubation with curcumin, the fluorescence intensity was reduced to a similar extent by both 5- and 16-SASL (62% and 64%, respectively) compared with liposomes without quenchers. In contrast, for non-PEGylated DMPC, 16-SASL was less effective than 5-SASL, reducing the signal to 78% versus 63% for 5-SASL. This indicates that curcumin penetrates both types of DMPC membranes and is oriented perpendicular to the bilayer surface; however, PEG enhances curcumin penetration toward the membrane center. In POPC membranes, both PEGylated and non-PEGylated, quenching was significant at both positions, but slightly more pronounced near the 5th carbon than at the membrane center (fluorescence reduced to 55% vs. 71% in PEGylated POPC, and 46% vs. 58% in non-PEGylated). These results indicate that even in unsaturated membranes, curcumin predominantly adopts a transbilayer orientation, although a fraction of the molecules may reside closer to the membrane surface, consistent with the carpet model [33]. In POPC membranes, which contain a double bond between the 9th and 10th carbon atoms in one acyl chain, this structural “defect” may hinder a fully parallel alignment of curcumin within the bilayer and promote its partial relocation toward the membrane surface. Because POPC comprises one saturated and one unsaturated chain, curcumin may still align parallel to the saturated chain. The use of 1,2-dioleoyl-*sn*-glycero-3-phosphocholine (DOPC) membranes, in which both acyl chains are unsaturated, should further test and validate this interpretation. Interestingly, POPC curcumin’s partition was not increased by PEG. The enhanced curcumin penetration into DMPC membranes in the presence of PEG is consistent with the calculated K_d_ values, which indicate that PEGylated DMPC binds curcumin to the greatest extent. Overall, fluorescence experiments, including curcumin binding and quenching studies, demonstrate that curcumin not only associates with liposomal membranes but also penetrates and fully spans the lipid bilayer, with PEGylated DMPC liposomes exhibiting the most uniform distribution of curcumin within the membrane. A more comprehensive understanding of fluorescence quenching mechanisms and efficiencies, as well as fluorophore accessibility and environmental heterogeneity, can be achieved through fluorescence lifetime measurements. As these aspects cannot be reliably inferred from steady-state fluorescence data, lifetime measurements will be pursued in future investigations.

### 4.2. EPR Experiments

In our previous work we demonstrated that curcumin modulates membrane properties in a concentration-dependent manner [11]. Therefore, we expected that PEG-induced increase in curcumin partitioning to lipid bilayer will enhance the effect of curcumin on bilayer properties. To explore this further, we conducted a series of experiments using EPR spectroscopy to investigate curcumin’s effects on lipid order, local polarity, and oxygen transport in PEGylated and non-PEGylated DMPC and POPC membranes.

The conventional continuous-wave (CW) EPR technique was used to investigate the mobility of polar headgroups, order parameters of lipid alkyl chains, rotational correlation times and polarity, whereas the pulse EPR technique, saturation recovery (SR), was employed to acquire the oxygen transport parameters.

The polar headgroup region of DMPC and POPC membranes was investigated using T-PC spin labels. In DMPC, the changes in the spectral parameters of T-PC, especially the linewidth of the low-field line (dh+), indicate that there is a slight increase in lipid headgroup mobility in the presence of curcumin, which is more visible in non-PEGylated membranes. In POPC the effects are negligible. The increase in lipid headgroup mobility is most probably caused by their separation by terminal rings of curcumin molecules containing polar hydroxyl and methoxyl groups [11]. Similar effects were observed for other membrane modifiers containing ring structures, such as cholesterol or xanthophylls [34,35,36]. Also, these effects were more pronounced in saturated membranes than in unsaturated ones, which may explain the minimal impact observed in POPC. The thickness of the membrane compared with the length of the curcumin molecule may also matter. In a thicker membrane, such as POPC, curcumin may sink deeper into the lipid part (Figure 7), which results in its smaller effect on polar headgroups. Similarly, unlike its effect on the motional freedom of DMPC polar headgroups, curcumin was previously found to have no impact on the thicker DSPC membrane [11].

The alkyl chain order and lipid mobility in DMPC and POPC membranes were investigated using 5- and 16-PC spin labels which probe regions near the lipid polar headgroups and at the membrane center, respectively. As already observed previously [11], at 25 °C curcumin increases the values of the S parameter in both regions of the DMPC membrane (from 0.63 to 0.66 at the 5th position, and from 0.13 to 0.18 at the 16th position). At 37 °C the effect is also observed, although the difference is smaller. The ordering effect of curcumin in DMPC seems to be slightly enhanced by PEG, particularly at 25 °C, whereas the effect of PEG alone is negligible. In POPC, curcumin’s ordering effect is observed only at the 5th position, and PEG does not further enhance this effect. This suggests that curcumin partitions to the DMPC membrane more efficiently when PEG is present, which is consistent with fluorescence data. A similar conclusion can be drawn from the analysis of the 16-PC rotational correlation time data. Both τ_2B_ and τ_2C_ values are higher in the presence of curcumin in both DMPC and POPC membranes; however, the effects are stronger in DMPC. Additionally, in the membranes containing curcumin there is a bigger difference between τ_2B_ and τ_2C_, which indicates that the motion of lipid alkyl chains is more anisotropic in the presence of curcumin than in the membranes without it. The ordering effect of curcumin, as reflected in correlation times, has already been demonstrated in DMPC [11]. Here, we confirmed this effect and demonstrated it also in unsaturated membranes (POPC) as well as in PEGylated membranes. At lower temperatures (25 °C in DMPC and 20 °C in POPC), the effect of curcumin on correlation times is more pronounced than at 37 °C. As previously shown, the impact of various compounds on lipid motion decreases with increasing temperature [11,22,34,35]. PEG alone exerts a slight but noticeable ordering effect in the membrane center, making it difficult to determine whether it further enhances curcumin’s effect. Nonetheless, the effects remain stronger in DMPC than in POPC membranes, consistent with the observations above. Regarding the effect of PEG alone, correlation times were the only parameters in our study that revealed a direct impact of PEG on lipid mobility. Lemaalem et al. investigated how PEGylation affects the mechanical properties of the lipid bilayer. Their MD simulations showed that PEGylated membranes possess increased bending rigidity and reduced lateral diffusion and explained that the mobility of lipids in PEGylated membranes was reduced due to PEG-induced crowding and increased interfacial viscosity [37]. Giakoumatos et al. studied colloid-supported lipid bilayer ordering via differential scanning calorimetry and fluidity using fluorescence recovery after photobleaching (FRAP) and found that up to 5 mol % of PEGylated lipids could be incorporated into the studied membranes without any pronounced effects. However, the fluorescence recovery of the more fluid DOPC membrane was markedly decelerated upon incorporating 10 mol % of PEGylated lipids, whilst insertion of the anchoring lipids (DOPE and DSPE without PEG2000) had no detectable impact. Therefore, they concluded that the quantity of incorporated PEG stabilizers, rather than the chemical nature of the lipid anchor, should be carefully optimized to ensure adequate colloidal stability without compromising membrane dynamics [38]. However, the results on flat bilayers immobilized on silica support cannot be fully compared to liposomes in suspension.

It is well established that compounds that alter membrane fluidity can also influence its polarity. In our previous work we studied the effect of curcumin on polarity of DMPC and DSPC membranes, and we showed that curcumin significantly changes the polarity profiles across these membranes, making them more water permeable [11]. Considering PEG’s hydrophilic nature, in the present work we investigated how PEG affects the polarity profiles of saturated (DMPC) and unsaturated (POPC) membranes, and how it modulates the curcumin-induced increase in polarity. We used 2A_z_ as a measure of local membrane polarity, which is directly obtained from the EPR spectra of spin labels in frozen liposome suspensions [25]. As expected, curcumin increases polarity within both membranes; however, in DMPC the effect is more pronounced in the polar headgroup region, whereas in POPC, this occurs in the membrane center. Interestingly, PEG alters curcumin’s effect on DMPC polarity—near the fifth position, curcumin’s impact is reduced compared to its effect in the absence of PEG. In the membrane center, PEG alone affects local polarity, making the membrane less polar (2A_z_ value in PEGylated DMPC is 67.5 G compared to 68.25 G in non-PEGylated one). A similar effect of PEGylated lipids on membrane polarity was shown by Aloia and Bartucci in dipalmitoylphosphatidylcholine (DPPC) membranes [39]. They reported sigmoidal water penetration and polarity profiles in sterically stabilized liposomes (SSLs) formed with submicellar amounts of 1,2-dipalmitoyl-sn-glycero-3-phosphoethanolamine-N-[methoxy(polyethylene glycol)-2000] (16:0-PEG2000-PE) incorporated into DPPC, and suggested that, compared to DPPC bilayers, SSLs exhibit increased hydrophobicity at both the polar/apolar interface and the chain termini [39]. In POPC, however, we observed the opposite effect of PEG on membrane polarity: PEG clearly reduces the hydrophobicity barrier and increases polarity throughout the membrane. At both positions, 5th and 16th, the 2A_z_ values are higher in the PEGylated POPC than in the non-PEGylated one (69.31 G vs. 68.9 G for 5-PC and 68.35 G vs. 67.5 G for 16-PC, respectively). The effect of curcumin on the polarity profile in POPC is significant in the membrane center. The pronounced effects of curcumin and PEG can be attributed to the higher hydrophobicity of POPC, as an unsaturated membrane, compared to DMPC [25]. Also, the polarity profiles in our study differ between DMPC and POPC, with POPC exhibiting a higher hydrophobicity barrier (2A_z_ values are lower at all positions across the POPC membrane compared to the DMPC membrane). As a result, curcumin may have a more pronounced effect on the 2A_z_ parameter, making the difference more noticeable. Also, because PEG clearly increases the polarity across the membrane, curcumin’s effect is less noticeable in the presence of PEG. Apart from its modest impact on correlation times, polarity seems to be another membrane feature changed by PEG alone. However, because the effect varies with the host lipid, drawing a definitive conclusion is difficult. In the polar headgroup region, the effect of curcumin on water accessibility is very strong in DMPC, which proves that curcumin effectively separates the lipid headgroups from each other. This is also consistent with the curcumin’s effect on T-PC parameters measured at room temperature, which was more pronounced in DMPC. Considering the structure of the curcumin molecule, the polarity profiles support its transbilayer orientation in all the membranes studied. Assuming this orientation, the effect of increased water penetration is observed in the places where the terminal rings containing polar hydroxyl and methoxyl groups of curcumin are located (Figure 7). A comparison of the curcumin’s effects on the polarity profiles of DMPC and POPC membranes allows us to conclude that the membrane thickness and unsaturation does not affect curcumin’s orientation within the membrane. Notably, the pronounced effect of curcumin on polarity at the center of the POPC membrane suggests that hydrogen bonding between two curcumin molecules spanning opposite leaflets is strong enough to stabilize the dimer, even when the membrane thickness and curcumin dimer length do not perfectly match. This observation is consistent with our previous comparison of DMPC and DSPC polarity profiles [11] and with the fluorescence quenching data obtained in the present study.

The final membrane characteristic obtained from EPR measurements was the oxygen transport parameter (OTP). Previous studies have demonstrated that the oxygen collision rate—a product of the local concentration and diffusion coefficient of molecular oxygen within the membrane—provides a highly sensitive measure of free volume within lipid bilayers. This free volume can be extremely small, often just large enough to accommodate a single oxygen molecule [40]. Due to its small size and suitable hydrophobicity, molecular oxygen can penetrate these transient, nanoscale voids in the membrane [19]. Consequently, the collision rate between oxygen and nitroxide spin labels at defined membrane positions reflects not only the dynamics of *gauche–trans* isomerization of lipid acyl chains but also the structural packing and conformational flexibility of neighboring lipids [40]. Our data show that curcumin significantly reduces the oxygen transport parameter at all locations in all membranes studied, with the effect being more pronounced in DMPC. This indicates that curcumin reduces the dynamics of *gauche-trans* isomerization of lipid hydrocarbon chains which is consistent with the data on order parameter and correlation times. Interestingly, curcumin also reduces OTP in the polar headgroup region, even though our other data indicate increased mobility and greater headgroup separation in this region. A similar effect has been observed for cholesterol, which markedly reduces OTP in the polar headgroup region of lipid bilayers [40] while simultaneously increasing water penetration into this region [25]. It was found that incorporation of cholesterol into lipid bilayers greatly reduced the oxygen collision rate at the place where cholesterol’s rigid steroid ring structure was located. Assuming a transbilayer location of curcumin, as suggested by Duda et al. [11] and confirmed in this work through quenching experiments and polarity profiles, it is likely that curcumin’s rigid rings positioned in the headgroup region as well as at the membrane center create a rigidity barrier that limits the penetration of small hydrophobic molecules such as oxygen. The effect of curcumin on the OTP in POPC membranes is smaller, which is consistent with the negligible impact of curcumin on other parameters describing membrane fluidity, such as the order parameter, correlation times and the mobility of polar headgroups in this membrane. PEG alone has no significant effect on OTP, nor does it noticeably alter curcumin’s impact in either membrane.

It is worth comparing the polarity and OTP profiles, as curcumin clearly alters both—but in opposite directions. While curcumin increases water penetration into the membrane, it simultaneously decreases oxygen penetration. Thus, curcumin appears to reduce the hydrophobicity barrier for polar molecules while increasing the rigidity barrier for apolar molecules. Both effects are more pronounced in a saturated DMPC membrane. PEG alone influences polarity profiles, as discussed above, but has no effect on oxygen penetration. This suggests that OTP reflects the rigidity barrier, which is not affected by PEG, as further supported by the absence of PEG’s effect on the order parameter.

The rigidity barrier formed by curcumin, which also reduces oxygen diffusion within the membrane, may be important for liposome stability. Membranes containing curcumin are not only ordered but also have lower oxygen content, limiting the potential for reactive oxygen species formation and thereby helping to prevent lipid peroxidation.

In the final discussion of curcumin’s effects on PEGylated and non-PEGylated membranes, it is important to consider concentration dependence. Numerous studies have demonstrated that curcumin’s impact on lipid membranes is strongly concentration-dependent. For example, in DOPC bilayers, curcumin exhibits a biphasic effect: it reduces water permeability at lower concentrations (≤~2 mol %) but increases permeability at higher concentrations, accompanied by altered membrane packing and indications of phase separation at ≥~3 mol % [10]. Similarly, fluorescence studies in DMPC membranes indicate that curcumin is homogeneously distributed at low mol fractions but forms fluidizing domains at higher concentrations (>1 mol %) [41]. In our previous work, we analyzed the effect of curcumin on DMPC and DSPC membranes at two concentrations (5 and 10 mol %) and observed a rigidifying effect at both levels, which was more pronounced at 10 mol % than at 5 mol % [11]. Concentrations above 10 mol % are generally discouraged due to the risk of phase separation and excessive disruption of acyl chain organization. Despite extensive literature on curcumin–membrane interactions, there remains no consensus on the precise molecular mechanisms underlying its concentration-dependent effects, including its membrane location and orientation. Nevertheless, for effective liposomal delivery, curcumin should be present at the highest feasible concentration to ensure efficient loading and optimal bioavailability.

## 5. Conclusions

Curcumin effectively interacts with liposome membranes, both saturated (DMPC) and unsaturated (POPC). PEG enhances the curcumin–lipid interaction, particularly in DMPC membranes, although it does not significantly alter the overall biophysical properties of the membrane. Curcumin penetrates deeply into the membrane, adopting a perpendicular orientation relative to the membrane surface, and in DMPC—especially when PEGylated—it reaches greater depths. The presence of curcumin increases lipid order and substantially enhances water penetration across the membrane, while simultaneously reducing oxygen diffusion at all membrane locations. Overall, our results suggest that PEGylated DMPC exhibits the most favorable biophysical properties for curcumin association and may represent a promising platform for curcumin delivery under the conditions tested.

## Figures and Tables

**Table 1:** Dissociation constants obtained from the global fitting of curcumin interaction with POPC, POPC-PEG, DMPC, and DMPC-PEG liposomes.

Samples	K_d_ * (µM)
POPC	270 ± 89
POPC-PEG	156 ± 46
DMPC	263 ± 58
DMPC-PEG	88 ± 21

* Values represent the best-fit parameters ± standard errors (SE) obtained from global fitting using the DynaFit program.

**Table 2:** Order parameter S of 5- and 16-PC in DMPC and POPC membranes, at 25 and 37 °C, in the presence and absence of 10 mol % curcumin and/or 7 mol % PEG.

Lipid Mixture	5-PC		16-PC	
	25 °C	37 °C	25 °C	37 °C
DMPC	0.63 ± 0.005 *	0.54 ± 0.05	0.13 ± 0.00	0.11 ± 0.00
DMPC-PEG	0.64 ± 0.005	0.54 ± 0.05	0.13 ± 0.00	0.10 ± 0.00
DMPC-CUR	0.66 ± 0.005	0.55 ± 0.01	0.18 ± 0.00	0.115 ± 0.005
DMPC-PEG-CUR	0.69 ± 0.01	0.57 ± 0.005	0.17 ± 0.01	0.10 ± 0.00
POPC	0.635 ± 0.005	0.57 ± 0.00	0.125 ± 0.005	0.105 ± 0.005
POPC-PEG	0.645 ± 0.005	0.555 ± 0.005	0.13 ± 0.00	0.10 ± 0.00
POPC-CUR	0.66 ± 0.00	0.585 ± 0.005	0.135 ± 0.005	0.105 ± 0.005
POPC-PEG-CUR	0.675 ± 0.005	0.585 ± 0.005	0.135 ± 0.005	0.10 ± 0.00

* Values represent the calculated parameters ± standard errors (SE).

## Data Availability

Data will be available from the corresponding author on request or publicly at https://ruj.uj.edu.pl/home (Jagiellonian University Repository).

## Supplementary Materials

The following supporting information can be downloaded at https://www.mdpi.com/article/10.3390/membranes16040137/s1. Figure S1: EPR spectra of 5-,16-PC, and T-PC spin labels in DMPC liposome obtained at 25 °C, Figure S2: EPR spectrum of 5-PC spin label in DMPC liposomes obtained at 120 K. A measured parameter (2A_z_) is indicated.
