# Supplementary material for: Curcumin–Lipid Interactions in PEGylated vs. Conventional Liposomes: A Combined Fluorescence and EPR Study
Source: Membranes (Basel). 2026 Apr 1;16(4):137. doi: 10.3390/membranes16040137 (PMC13117882; doi:10.3390/membranes16040137)
Supplement: Supplementary file 1 [file membranes-16-00137-s001.zip › membranes-4030379-supplementary.pdf]

Supplementary Materials

S parameter values were calculated using the following formula [Marsh, 1981]:

$$S = 0.5407 * \frac{A_{II} - A_{\perp}}{a}$$

where A_{II} and $A_{\perp}$ are hyperfine splitting values measured as the distances between outer and inner EPR spectrum extrema, respectively, and *a* is an isotropic hyperfine splitting.

Rotational correlation times of 16-PC were calculated according to the formulas [Berliner, 1978]:

$$\tau_{2b} = 6.51 * 10^{-10} * \Delta W \left| (\frac{h_0}{h_{-1}})^{\frac{1}{2}} - (\frac{h_0}{h_{+1}})^{\frac{1}{2}} \right|$$

$$\tau_{2c} = 6.51 * 10^{-10} * \Delta W \left| (\frac{h_0}{h_{-1}})^{\frac{1}{2}} + (\frac{h_0}{h_{+1}})^{\frac{1}{2}} - 2 \right|$$

where ΔW is the linewidth of the central line of EPR spectrum, and h_0, h_{-1} and h_{+1} are the amplitudes of central, high-field and low-field spectral lines, respectively.

Fig. S1 demonstrates how the required spectral parameters were measured.

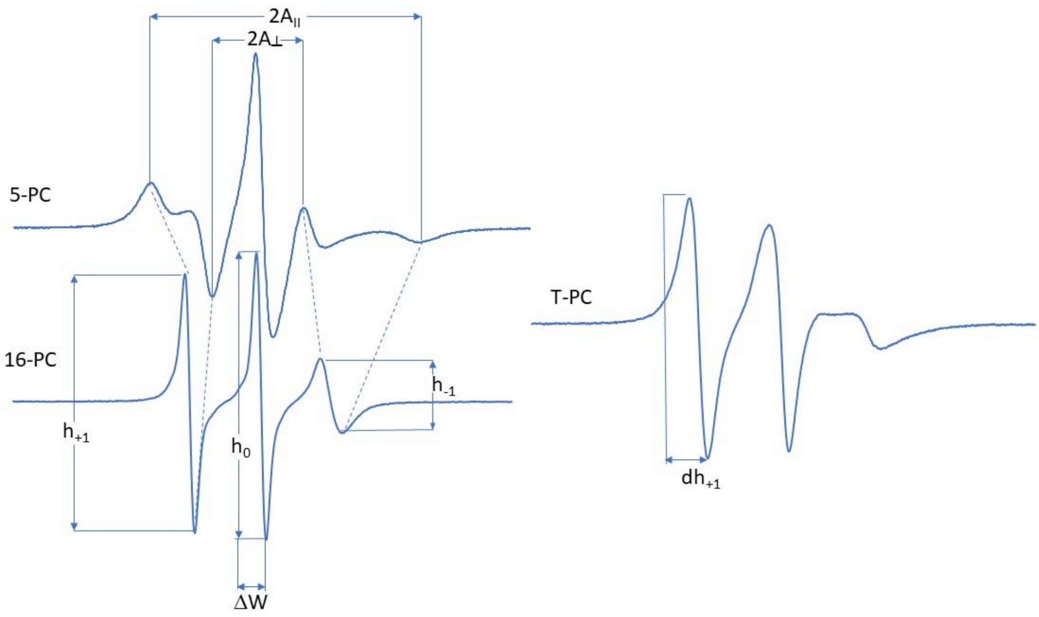

Figure S1. EPR spectra of 5-,16-PC, and T-PC spin labels in DMPC liposome obtained at 25 °C. Measured values are indicated: the peak-to-peak distances (2 A_{II} and 2$A_{\perp}$) used for calculating the order parameter S, the width of the central line (ΔW) and the amplitudes of all three lines of 16-PC spectra (h_0, h_{-1}, h_{+1}) used for calculating the rotational correlation times and the width of the lowfield line (dh_{+1}) of T-PC spectra. The amplitudes of the central and high-field lines of T-PC spectra were measured the same way as of 16-PC.

2Az (z-component of the hyperfine interaction tensor) used for polarity assessment was obtained directly from the spectra of spin labels in frozen liposome suspensions as indicated in Fig. S2.

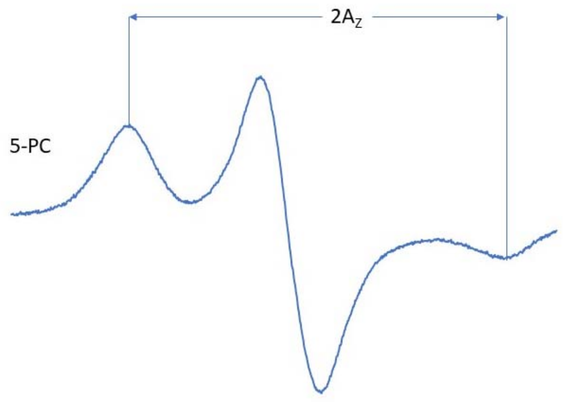

Figure S2. EPR spectrum of 5-PC spin label in DMPC liposomes obtained at 120K. A measured parameter (2Az) is indicated.

Marsh, D. Electron Spin Resonance: Spin Labels; 1981;

Berliner, L.J. Spin Labeling in Enzymology: Spin-Labeled Enzymes and Proteins. Methods Enzymol 1978, 49, 418–480;
